# Insight into the Mechanism of CO Oxidation on WO_3_(001) Surfaces for Gas Sensing: A DFT Study

**DOI:** 10.3390/s17081898

**Published:** 2017-08-17

**Authors:** Hua Jin, Hegen Zhou, Yongfan Zhang

**Affiliations:** 1 College of Chemistry and Biology Engineering, Yichun University, Yichun 336000, China; 999025@jxycu.edu.cn; 2 Engineering Center of Jiangxi University for Lithium Energy, Yichun University, Yichun 336000, China; 3 College of Chemistry, Fuzhou University, Fuzhou 350116, China; 4 State Key Laboratory of Photocatalysis on Energy and Environment, Fuzhou 350002, China

**Keywords:** tungsten trioxide, oxidation reaction, CO sensor, density functional theory

## Abstract

The mechanism of CO oxidation on the WO_3_(001) surface for gas sensing performance has been systematically investigated by means of first principles density functional theory (DFT) calculations. Our results show that the oxidation of CO molecule on the perfect WO_3_(001) surface induces the formation of surface oxygen vacancies, which results in an increase of the surface conductance. This defective WO_3_(001) surface can be re-oxidized by the O_2_ molecules in the atmosphere. During this step, the active O_2_^−^ species is generated, accompanied with the obvious charge transfer from the surface to O_2_ molecule, and correspondingly, the surface conductivity is reduced. The O_2_^−^ species tends to take part in the subsequent reaction with the CO molecule, and after releasing CO_2_ molecule, the perfect WO_3_(001) surface is finally reproduced. The activation energy barriers and the reaction energies associated with above surface reactions are determined, and from the kinetics viewpoint, the oxidation of CO molecule on the perfect WO_3_(001) surface is the rate-limiting step with an activation barrier of about 0.91 eV.

## 1. Introduction

Metal oxides are widely used as gas sensitive materials due to their reproducibility and typical surface properties which are suitable for gas detection. Tungsten trioxide (WO_3_) is one of the most promising gas sensor candidates. Up to now, most studies have focused on the application of WO_3_ in NO_x_ sensors [1,2,3,4,5], while little attention has been paid to its use in sensors for other gases. Carbon monoxide (CO) is a toxic and environmentally-hazardous gas. More than 80% of the CO in the atmosphere comes from the imperfect combustion of carbonaceous materials and vehicle exhaust. With the development of the urban environment and increased auto use, it is necessary to detect the amount of CO to control the air quality, and the development of CO sensing materials has become an important subject of current research [6,7,8,9,10,11,12]. Owing to their advantages of wide detection range, good stability, long lifetime and rapid response, metal oxides are absolutely competitive candidate materials for CO detection [13,14,15]. However, the microscopic mechanism of the surface reactions underlying the sensing properties toward CO are still far from being understood, especially for the WO_3_-based sensor materials.

Before 2006, Azad et al. [16] reported that WO_3_ has the sensitivity towards CO gas. Then Wu et al. [17] prepared a CoOOH-WO_3_ type CO sensor. Their experimental results showed that such a composite sensing material produces a better CO response at 25 °C. A mechanism of CO sensing on this nanocomposite surface has been supposed based on the adsorption and desorption of CO molecules, as well as the surface oxidation reactions between adsorbed CO and adsorbed oxygen atoms. Hübner [18] and co-workers examined the sensing of CO with WO_3_-based gas sensors as a function of the oxygen background conditions, which attracted our great interest and attention. They recorded CO_2_ formation when CO was exposed to the WO_3_ surface in spite of a very low oxygen partial pressure. This phenomenon indicates that the CO oxidation by the metal oxide material, that is to say, the direct reduction of the WO_3_ surface is the most possible cause for the decrease of surface resistivity, which is much different from the sensing mechanism observed in SnO_2_ sensor materials. Moreover, they observed the defective surface was re-oxidized and the surface resistance increased when the oxygen partial pressure increased due to the fact that the O_2_ molecules react with oxygen vacancies. Thus the magnitude of WO_3_ sensor signal depends on the equilibrium between the generation of oxygen vacancies and their cancellation. Ahsan and co-workers [19] synthesized Fe-doped WO_3_ thin films with good response to CO gas at 150 °C by a thermal evaporation method. Similarly to the findings above, the CO molecule is oxidized to CO_2_ on this nanostructure surface, resulting in a drop in film resistance, and the active species for CO oxidization is assumed to be O_2_^−^. Recently, a new-style WO_3_-based nanowire was successfully fabricated by Zappa’s group as a CO detector [20]. They deduced the change of surface resistance on account of the balance between the adsorbed active oxygen species (O_2_^−^, O^−^ or O^2^^−^) and their vanishing due to the chemical reaction with CO.

Generally speaking, the resistivity change of the sensors is associated with the oxidation-reduction reaction of gas adsorbed on the sensor surface, so understanding the oxidation reaction of CO on the surface is a key to improve the sensing performance of the sensor material, and carrying out corresponding theoretical research at the molecular level is very necessary. However, compared with the experimental reports, theoretical publications in regard to CO sensing with WO_3_ gas sensor are lacking so far. Most present theoretical works mainly focus on the adsorption behavior of CO at WO_3_ surfaces [21], while few studies refer to the CO oxidation on the WO_3_ surface for sensing. Oison [22] et al. investigated the CO adsorption condition on WO_3_ film with and without redox reaction via ab initio calculations. On the basis of their calculations, the former is much more important for the sensing mechanism than the latter. The number of oxygen vacancies ($V_{O}^{*}$) and surface conductivity increase when CO is oxidized to CO_2_ on the WO_3_ surface, which is in accordance with previous experimental results [18]. In recent studies, a series of first principle calculations have been performed by Tian’s group [23,24]. Their results further confirm the significance of CO oxidation process for WO_3_ sensing mechanism, and suggest that the existence of the oxygen vacancies decreased the sensitivity of WO_3_ surface towards CO to some extent.

As a surface-controlled type semiconductor, WO_3_ surface has many active sites. When the reduced CO gases approaches to the WO_3_ sensor surface, they can be oxidized by the surface oxygen species, but perhaps also by the lattice oxygen atoms of WO_3_ surface or the oxygen species (O_2_^−^, O^−^ or O^2^^−^) from the chemisorbed oxygen molecules in the atmosphere. Meanwhile, the chemical reactions between CO and WO_3_ surface are involved in the change of the oxygen vacancy concentration, which is directly related to the surface resistivity of the sensor material. It should be noted that although pure WO_3_ needs to be modified (doping, noble metal deposition, or modification of the morphology), or used at a specific temperature to improve its sensing performance, the study of CO sensing progress on a clean WO_3_ surface is still of great importance, as it could provide theoretical guidance for the development of better WO_3_-based sensors. Based on the above, in this work we have systematically discussed the oxidation reactions of CO molecule on the WO_3_(001) surfaces for gas sensing. Particularly, we reveal the role of oxygen vacancies and active oxygen species on the sensing performance of WO_3_-based materials.

## 2. Computational Details

First-principle calculations based on DFT were carried out utilizing the Vienna ab initio simulation package (VASP) [25,26,27,28], and the ultrasoft pseudopotentials [29,30] were used to describe the interaction between the ion cores and valence electrons. The generalized Perdew-Wang gradient approximation (PW91) [31] exchange–correlation functional was employed, and the kinetic cutoff energy for the planewave expansion was set to 400 eV. In the calculations, the convergence energy threshold for self-consistent iteration was set at 10^−4^ eV, and the residual atomic forces were smaller than 0.03 eV/Å. The effects of spin polarization were considered, and the dipole correction in the direction of the surface normal was applied.

**Figure 1:**
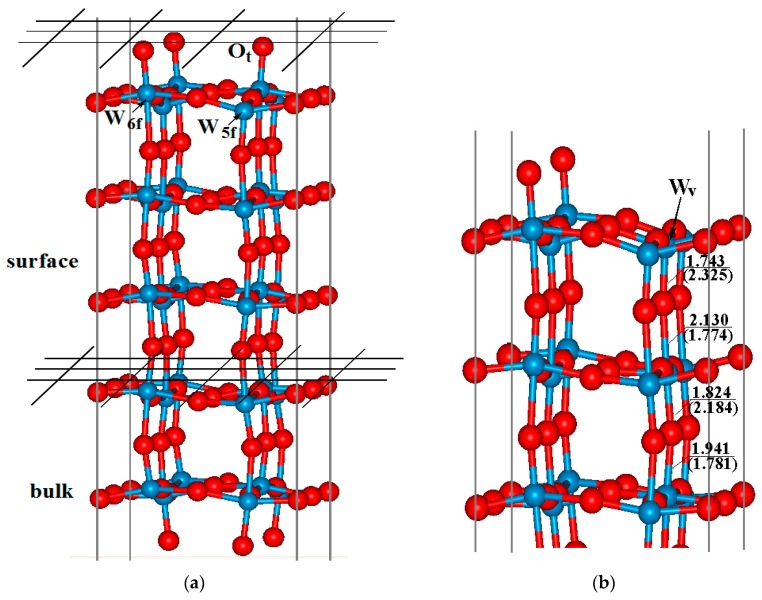
Configuration of (**a**) five-layer perfect WO_3_(001) surface and (**b**) defective WO_3_(001) surface with half of the top oxygen atoms missing. The blue and red balls stand for tungsten and oxygen atoms, respectively.

As in our previous study [32], a five-layer periodic slab model were adopted to simulated the WO_3_(001) surface with γ-monoclinic phase (Figure 1a), and in the calculations, half of the terminal oxygen atoms on the top layer were transferred to the bottom to avoid residual charges on the surface. During the structural optimization, the atoms on the top three layers were fully relaxed in all directions while the others were fixed to bulk. The vacuum gap was set to 10 Å for the investigation on the adsorption and oxidation of CO molecule. During the geometry optimization and energy calculations of free CO or O_2_, the molecule was placed in a 15 Å × 15 Å × 15 Å cubic box in order to avoid the interaction between the adjacent molecules. The optimized bond lengths of C–O and O–O bond are 1.144 Å and 1.237 Å, which are in good agreement with the experimental values and other theoretical results [22].

The adsorption energy (*E_ads_*) of CO or O_2_ molecule on the WO_3_(001) surface is described as:

(1) $$E_{ads}\;=\;E_{slab}\;+\;E_{molecule}\;-\;E_{total}$$

where *E_total_* is the total energy for the slabs with the adsorption of CO or O_2_, *E_slab_* is the total energy of the WO_3_(001) surface, and *E_molecule_* stands for the energies of free CO or O_2_. For the O_2_ molecule, the ground state is triplet state. The positive value of *E_ads_* indicates the adsorption is thermodynamically favorable.

Since the oxygen vacancy is also considered, the formation energy of oxygen vacancy (*E_vac_*) on the WO_3_(001) can be calculated according to the following formula [22]:

(2) $$E_{vac}\;=\;(E_{def}\;+\;\frac{1}{2}nE_{O_{2}}\;-\;E_{perf})/n$$

in which *E_def_*, *E_perf_* are the total energies of the WO_3_(001) surface with and without oxygen vacancy, respectively. $E_{O_{2}}$ is the total energy of free O_2_ molecule. *n* represents the number of oxygen atom removed from the surface. The negative value indicates the formation of oxygen vacancy is thermodynamically feasible.

Concerning the CO oxidation reactions on the WO_3_(001) surface, the climbing image nudged elastic band (CINEB) method [33,34] was used to determine the minimum energy path (MEP). The energy barrier of the CO oxidation reaction is defined by the energy difference between the transition state (TS) and reactant. The vibrational frequency analysis was also performed to ensure that the predicted TS corresponded to the first-order saddle point in the reaction path. The values of frequencies were calculated from the diagonalization of the mass-weighted Hessian matrix constructed by the finite-difference process.

## 3. Results and Discussion

### 3.1. Surface Oxygen Species on WO_3_(001) Surface

**Figure 2:**
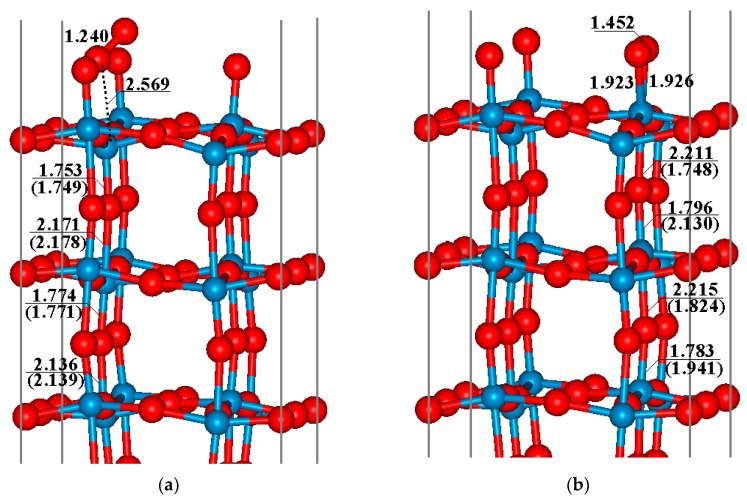
Optimized configurations of O_2_ adsorption on (**a**) perfect and (**b**) defective WO_3_(001) surface WO_3_(001) surface, respectively. The bond lengths (Å) near the adsorption site are given, and the data in the parenthesis are in relative to the value before O_2_ adsorption. Only the top three layers are shown in the figure.

One of the first problems to solve is the possible oxygen species for CO oxidation, which is significant in sensing. Therefore before exploring the mechanism of CO oxidization on the WO_3_(001) surfaces, we have examined the adsorption behavior of O_2_ on the sensor surface, including the perfect and defective WO_3_(001) surfaces, and the most stable optimized configurations are displayed in Figure 2a,b, respectively. With respect to the adsorption of O_2_ on the perfect WO_3_(001) surface (Figure 2a), the distance between the oxygen atom of O_2_ molecule (O_m_) and fivefold-coordinated tungsten atom (W_5f_) atom is 2.569 Å, which is obviously larger than the normal W–O single bond (about1.9~2.0 Å), and the length of O–O bond of oxygen molecule is 1.240 Å, being close to the value (1.237 Å) of a free oxygen molecule, so it can be expected that the interactions between O_2_ and the perfect WO_3_(001) are weak. Moreover, the structure of the WO_3_(001) surface changes slightly compared to the pristine surface (see the data shown in the parentheses in Figure 2), and the variations of the W–O bond lengths are smaller than 0.01 Å. The calculated adsorption energy of this configuration is −0.42 eV, indicating that the adsorption of oxygen molecule on the perfect WO_3_(001) surface is thermodynamically unfavorable. Further analysis of Bader charge reveals that there is nearly no change of surface conductivity after O_2_ adsorbing on the perfect WO_3_(001) surface (about 0.01 e electrons transfer from the oxygen molecule to surface).

For the adsorption of O_2_ molecule on the defective surface, the WO_3_(001) surface with half of the top oxygen atoms removed has been chosen as a theoretical model (Figure 1b). When the O_2_ molecule is adsorbed, several possible configurations have been taken into account and the optimized structures are provided in the supplementary materials (Figure S1). Among them, the most stable adsorption structure is that the O_2_ molecule occupies the site just above the tungsten atom that the top oxygen atom is removed (namely W_v_ for clarity), and the O–O bond is parallel to the surface (see Figure 2b). The adsorption energy of this configuration is about 1.10 eV. As presented in the figure, two oxygen atoms of O_2_ (O_m_) are simultaneously bonded with W_v_ atom, the length of W_v_–O_m_ bond is about 1.93 Å. Due to the obvious interaction between O_m_ and W_v_ atoms, the O–O bond of O_2_ molecule is activated and the bond length increases from 1.237 Å to 1.452 Å. After the adsorption of O_2_, there are about 0.7 e electrons transferred from the defective surface to the O_2_ moiety, which improves the surface resistivity. Hence, an O_2_^−^ species is formed when O_2_ is adsorbed on the defective WO_3_(001) surface, and such active group will play an important role in the CO oxidation. We have further investigated the reaction path for the adsorption of O_2_ molecule on the defective WO_3_(001) surface by using the CINEB method, and the results indicate that no energy barrier is obtained for this process. Therefore, it seems that when the defective WO_3_(001) surface is exposed to air, the O_2_ molecule can be easily adsorbed on the surface.

### 3.2. Adsorption of CO on WO_3_(001) Surfaces

**Figure 3:**
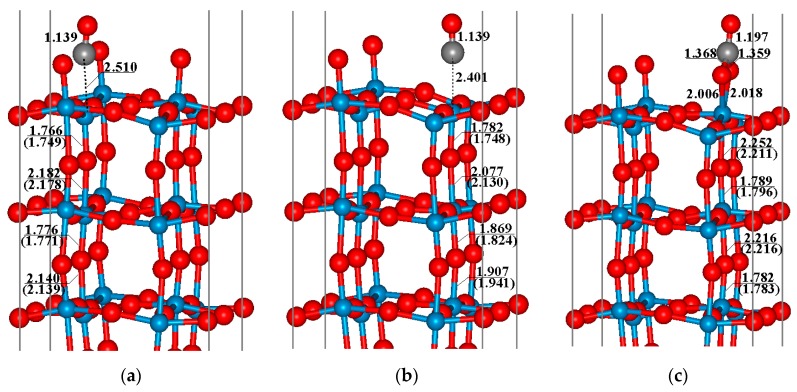
Adsorption structures of CO on (**a**) perfect WO_3_(001) surface; (**b**) defective WO_3_(001) surface, and (**c**) defective WO_3_(001) surface modified by the pre-adsorbed O_2_ molecule. The data in the parenthesis is corresponding to the value before CO adsorption.

In this section, we will discuss the adsorptions of CO molecules on the perfect, defective and O_2_ pre-adsorbed WO_3_(001) surface, respectively. Figure 3a displays the optimized structure for the adsorption of CO on the perfect WO_3_(001) surface. It can be seen that the C–W bond length is 2.510 Å and the distance between C and O atom (namely O_c_) in CO is nearly the same as that of the free CO molecule (1.139 Å vs. 1.144 Å), indicating a weak interaction between the CO molecule and surface. In addition, a slight shrink of the C–O bond demonstrates that the CO molecule acts as an electron donor when it interacts with the perfect WO_3_(001) surface, and this conclusion is in accordance with the electron affinity (EA) result obtained by Oison et al. [22]. As shown in Figure 3a, the adsorption of CO has a small effect on the surface structure, so it can be expected that the electronic structure of the perfect WO_3_(001) surface is maintained after CO adsorption, implying that the variation of the electric resistance of the system caused by CO adsorption is small. The result of adsorption energy (*E*_ads_) also shows that the CO molecule is physically adsorbed on the perfect WO_3_(001) surface with a small *E*_ads_ of 0.44 eV. This result is also in good agreement with the value (*E_ads_* = 0.37 eV) reported in a recent work by Oison et al. [22].

Similar to the perfect WO_3_(001) surface, the CO molecule also prefers to interact with the W_v_ atom just under the oxygen vacancy on the defective WO_3_(001) surface through its C ending. The adsorption energy of this configuration is calculated to be about 0.34 eV, which is slightly smaller than the value of perfect surface. Although the optimized W_v_–C bond length (2.401 Å) is about 0.1 Å shorter than that on the perfect surface, the interaction between CO and defective surface is still weak. Correspondingly, the adsorption of CO molecule also has little influence on the properties of the substrate, including the surface resistance.

For the defective WO_3_(001) surface that is modified by the pre-adsorbed O_2_ molecule, the most favorable adsorption configuration is quite different from above two cases. As presented in Figure 3c, the CO molecule directly interacts with the pre-adsorbed O_2_ (namely the O_2_^−^ species), in which the C atom is connected with two O atoms of O_2_ molecule. The predicted length of C–O_m_ bond is about 1.36 Å, and the O_m_–O_m_ bond is broken. Furthermore, a carbonate-like compound is formed except that the C–O_m_ bond is somewhat longer. After the adsorption of CO molecule, the W_v_–O_m_ bonds elongate from about 1.9 Å to 2.0 Å. The formation of this adsorption structure is quite exothermic with an adsorption energy of 3.07 eV, and therefore, the strongly thermodynamic driving force for the formation of this configuration can be expected.

### 3.3. Oxidation of CO on WO_3_(001) Surfaces

**Figure 4:**
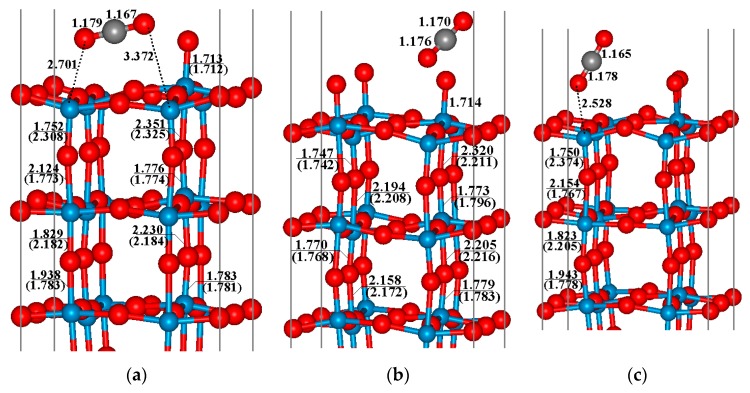
Oxidation product for CO reacting with (**a**) the O_t_ atom on perfect WO_3_(001) surface; (**b**) the O atom belongs to the O_2_^−^ species and (**c**) the O_t_ atom on defective WO_3_(001) surface modified by the pre-adsorbed O_2_ molecule.

**Figure 5:**
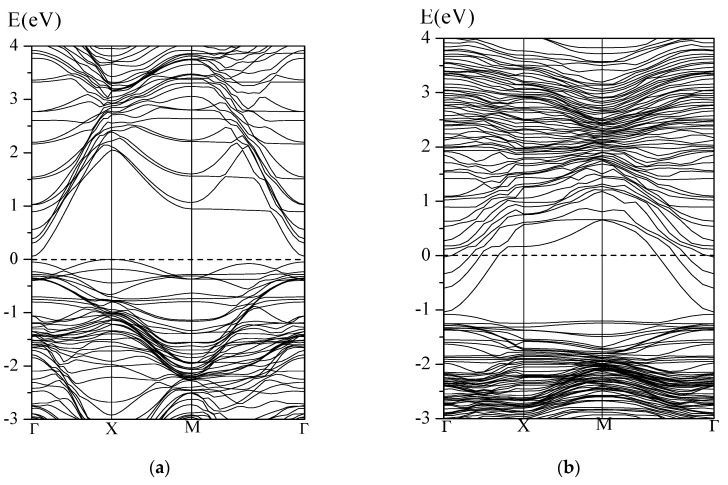
Band structures of the (**a**) perfect and (**b**) defective WO_3_(001) surface.

On the basis of the previous discussion, the lattice oxygen and oxygen species O_2_^−^ are the main active sites for the redox reactions between CO gas and the WO_3_ sensor surface. When CO attacks the perfect WO_3_(001) surface, a top oxygen (O_t_) transfers to the molecule, and a CO_2_ product is produced. From Figure 4a, the lengths of C–O_t_ and C–O_c_ bond lengths are 1.179 Å and 1.167 Å, respectively, very close to the length of C–O bond in the free CO_2_ molecule (1.16 Å). The newly formed CO_2_ molecule is weakly adsorbed on the surface with the distance between O and surface W atoms longer than 2.7 Å. It is noted that, accompanying the generation of CO_2_ molecules, the perfect WO_3_(001) surface becomes defective, and such oxygen vacancy leads to significant changes of the surface structure (see Figure 4a). Comparing the band structures between the perfect and defective WO_3_(001) surfaces given in Figure 5, a great influence for the electronic property can be observed when the oxygen vacancy is formed. It is clear that the semiconductor property of WO_3_ bulk is preserved for the perfect surface although the band gap is small due to the well-known shortcoming of pure DFT method. The removing of the top oxygen atom leads to several partly occupied energy bands appeared at the Fermi level, resulting in the metallic character of the defective WO_3_(001) surface. Therefore, it can be expected that the conductivity of the WO_3_(001) surface is enhanced after oxidation of CO molecule.

The oxidation reaction of CO on the perfect WO_3_(001) surface can be described as:

(3) $$W_{n}O_{3n}\;+\;\mathrm{CO}\;\to \;W_{n}O_{3n-1}\;+\;V_{O}^{}\cdot \;+\;e\;+{\;\mathrm{CO}}_{2}$$

leading to the formation of oxygen vacancy ($V_{O}^{}\cdot$) and releasing of electron. Accompanying the increase of the oxygen vacancy concentration, a sharp change in the electronic conductivity is achieved. The corresponding reaction energy of CO oxidation that is defined as the energy difference between before and after CO oxidation is about 1.50 eV, indicating that such a process is exothermic.

In the case of CO oxidation on the WO_3_(001) surface which is modified by pre-adsorbed oxygen, both oxygen species on this surface, namely the top oxygen and the oxygens belonging to the O_2_^−^ group may react with the CO molecule. The corresponding optimized structures have been given in Figure 4b,c, respectively. Similar to a perfect surface, when CO reacts with the lattice oxygen (O_t_), the CO_2_ product is generated and a new oxygen vacancy is created, while for the case of the O_2_^−^ group, one O atom of the active oxygen species transfers to CO and the CO_2_ product is physically adsorbed on the surface. It is interesting that after releasing CO_2_ molecule, the perfect WO_3_(001) surface is reproduced since one oxygen atom occupies the vacancy site. Contrasting the total energies of these two systems, the latter is about 2.93 eV more stable than the former. Hence, CO tends to react with the active oxygen species O_2_^−^ for the O_2_ pre-adsorbed WO_3_(001) surface, which is in conformity with the experimental results reported by Hübner et al. [18]. Accordingly, the CO oxidation process on this modified surface can be described by following reaction:

(4) $$W_{n}O_{3n-1}\;+\;\mathrm{CO}\;+{\;O}_{2}\to {\;W}_{n}O_{3n}\;+{\;\mathrm{CO}}_{2}$$

Therefore, after reacting with CO, the CO_2_ molecule is yielded and the perfect WO_3_(001) surface is reproduced. In addition, the reaction energy of above reaction is strongly exothermic by 4.25 eV.

### 3.4. Mechanism of CO Oxidation on WO_3_(001) Surfaces

**Figure 6:**
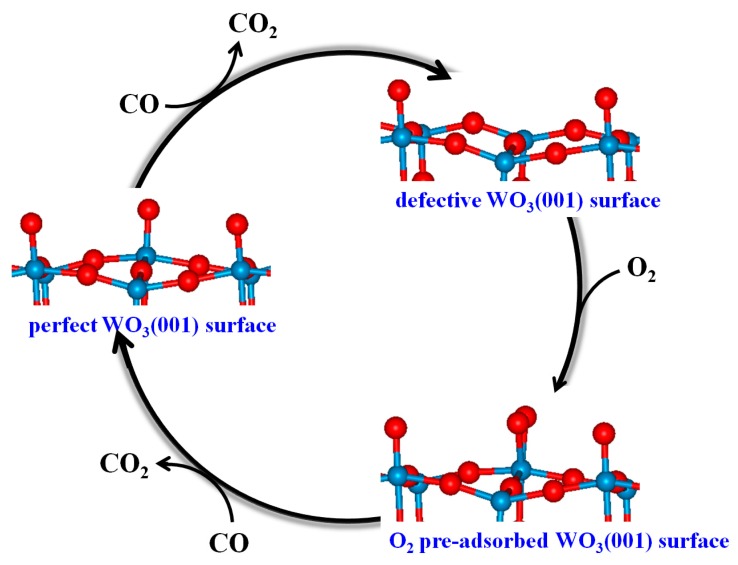
Schematic representation of a possible full sensing cycle of the WO_3_(001) surface as a CO sensor.

In summary, a possible full oxidation cycle can be proposed for the WO_3_(001) surface as a CO sensor. As can be seen from the schematic representation of the catalytic cycle shown in Figure 6, during the oxidation of CO on the perfect WO_3_(001) surface, the surface terminal oxygen (O_t_) is consumed along with the generation of CO_2_. At the same time, the WO_3_(001) surface becomes defective, which causes a decrease of the resistivity. When the defective WO_3_(001) surface is exposed to air, the O_2_ molecule prefers to be adsorbed on the vacancy site and then the active O_2_^−^ species is formed on the surface. Due to the electron transfer from the surface to the molecule, the surface resistance is enhanced. Finally, the surface active oxygen species (O_2_^−^) reacts with CO, and after releasing of the CO_2_, the original WO_3_(001) surface is regenerated and the electronic signal is recovered.

**Figure 7:**
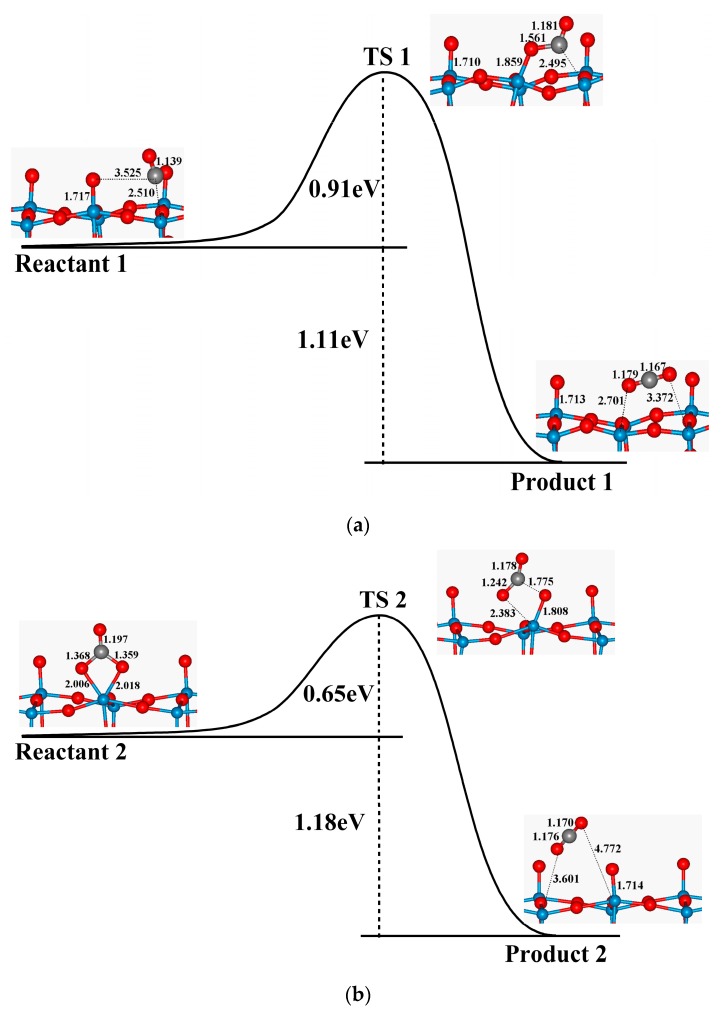
Minimum energy paths (MEP) for CO oxidation on (**a**) the perfect and (**b**) the O_2_ pre-adsorbed WO_3_(001) surface. The configurations of the top layer and some bond distances (Å) of the initial and final states, as well as the transition state (TS) are also shown in the figures.

Actually, the total oxidation process can be decomposed into three steps: (1) CO oxidation on the perfect WO_3_(001) surface and the formation of the defective WO_3_(001) surface; (2) the creation of the O_2_^−^ active species; (3) CO oxidation on the O_2_-preadsorbed WO_3_(001) surface and the regeneration of the original WO_3_(001) surface. All these steps are extensively exothermic and seem to be thermodynamically feasible. However, which is the rate-limiting step? In order to answer this question, we have carried out additional calculations to obtain the minimum energy paths (MEP) of the overall process. The calculated energy profiles for oxidation of CO on the perfect and O_2_-preadsorbed WO_3_(001) surfaces by using CINEB method are presented in Figure 7.

For the perfect WO_3_(001) surface (Figure 7a), a transition state (TS1) is identified with an energy barrier of 0.91 eV, and the vibrational frequency calculation shows that there is only one imaginary frequency (273 i·cm^−1^) for this configuration. As for TS1, the CO molecule bonds with O_t_ atom, and the length of C–O_t_ bond is 1.561 Å. As a result of the formation of C–O_t_ bond, the configuration of the WO_3_(001) surface is changed remarkably, especially the bond between the six-coordinated tungsten atom (W_6f_) and the terminal oxygen atom (O_t_) is elongated to 1.859 Å, which is about 0.14 Å longer than that in the initial state. Because of the considerable variation of the surface geometry, an obvious energy barrier is required to produce CO_2_ on the perfect surface. While concerning on the O_2_^–^ preadsorbed WO_3_(001) surface, the initial state is corresponding to an intermediate that the C atom of CO is bonded with two O atoms of O_2_^−^, as mentioned in Section 3.2. The CO_2_ molecule can be generated from this configuration, and meanwhile the perfect WO_3_(001) surface is reproduced. The transition state (TS2) of this process is shown in Figure 7b, in which one of the W–O_m_ bond tends to be broken and the corresponding bond length is enlarged to 2.383 Å; in the meantime, the length of one C–O_m_ bond decreases from 1.368 Å to 1.242 Å, while the length of another C–O_m_ bond increases to 1.775 Å. The energy barrier is calculated to be 0.65 eV, and the vibrational frequency calculation demonstrates that there is only one imaginary frequency (387 i·cm^−1^) for this transition state. Compared the heights of the energy barrier of those two steps, it seems that the CO oxidation on the perfect WO_3_(001) surface is the rate-determining step from the kinetics viewpoint.

## 4. Conclusions

In this work, the oxidation reactions of CO molecule on the WO_3_(001) surfaces for CO sensing have been systematically investigated by DFT calculations. Owing to the formation of active oxygen species at the surface and the generation of the surface oxygen vacancies, the oxidation of CO on the WO_3_(001) surface results in obvious variations of the electronic properties which are directly related to the change of the resistance of WO_3_ sensor. Our results suggest that the adsorption of O_2_ molecule on the perfect WO_3_(001) surface is thermodynamically unfavorable and has little effect on the surface resistance, while a strong chemisorption of O_2_ occuring on the defective surface is predicted. The O_2_ molecule adsorbed at the vacancy site leads to the formation of an important oxygen species (namely O_2_^−^) for further CO oxidation reactions, while also enhancing the surface resistivity. We have shown that CO is oxidized to CO_2_ by the top oxygen atom for the perfect WO_3_(001) surface, resulting in the formation of a defective surface with oxygen vacancy at surface and the reduction of surface resistivity. However, concerning the CO oxidation on the O_2_-preadsorbed WO_3_(001) surface, CO prefers to taking away one O atom of the active O_2_^−^ species instead of the top oxygen. Simultaneously, the pristine WO_3_(001) surface is regenerated and the surface resistance recovered. The overall oxidation process can be summarized as: (a) CO oxidation on the perfect WO_3_(001) surface and the formation of the defective WO_3_(001) surface; (b) the formation of the O_2_^−^ active species; (c) CO oxidation on the O_2_-preadsorbed WO_3_(001) surface and the regeneration of the perfect WO_3_(001) surface. From the kinetics point of view, the CO oxidation on the perfect WO_3_(001) surface is the rate-limiting step.

## Supplementary Materials

The following are available online at http://www.mdpi.com/1424-8220/17/8/1898/s1, Figure S1: Possible adsorption configurations for O_2_ adsorption on the defective WO_3_(001) surface. The bond lengths (Å) close to the adsorption site are given, and the data in the parenthesis are in relative to the defective WO_3_(001) surface before O_2_ adsorption. In addition, the corresponding adsorption energies have also been provided.
