# Supplementary material for: Insight into the Mechanism of CO Oxidation on WO3(001) Surfaces for Gas Sensing: A DFT Study
Source: Sensors (Basel). 2017 Aug 17;17(8):1898. doi: 10.3390/s17081898 (PMC5579943; doi:10.3390/s17081898)
Supplement: Supplementary file 1 [file sensors-17-01898-s001.pdf]

Supplementary Materials

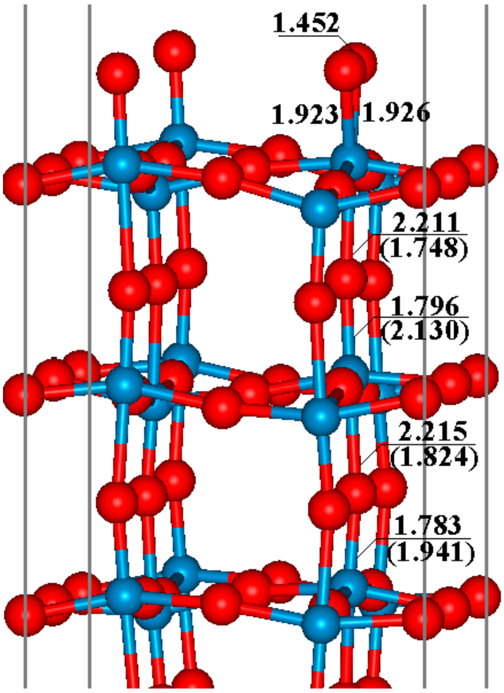

1.110eV

(a)

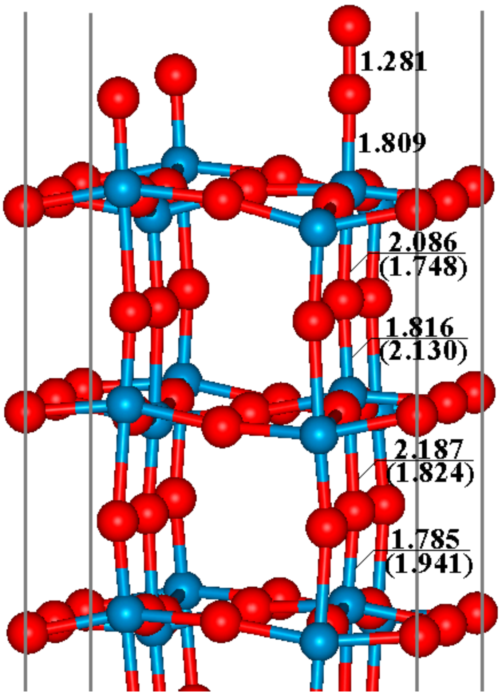

0.450eV

(b)

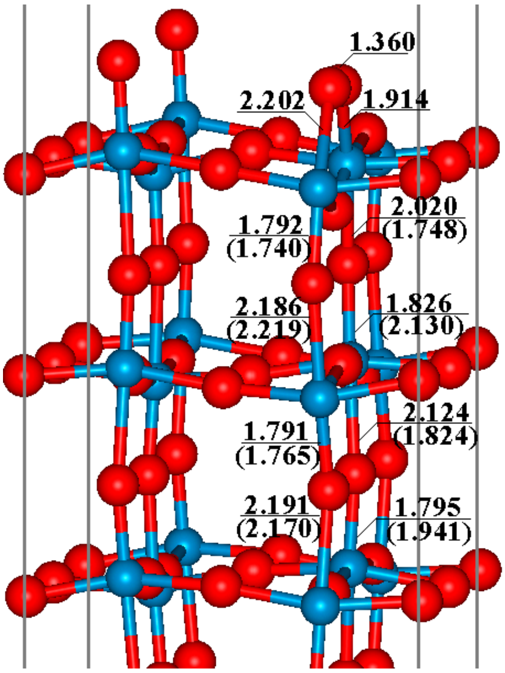

0.378eV

(c)

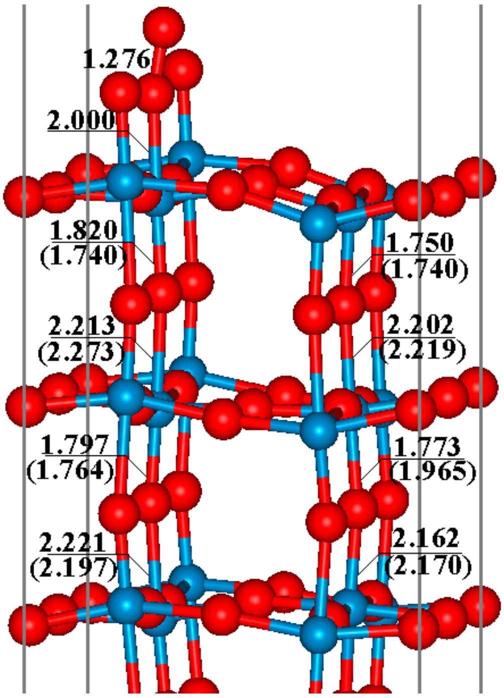

-0.597eV

(d)

Figure S1. *Cont.*

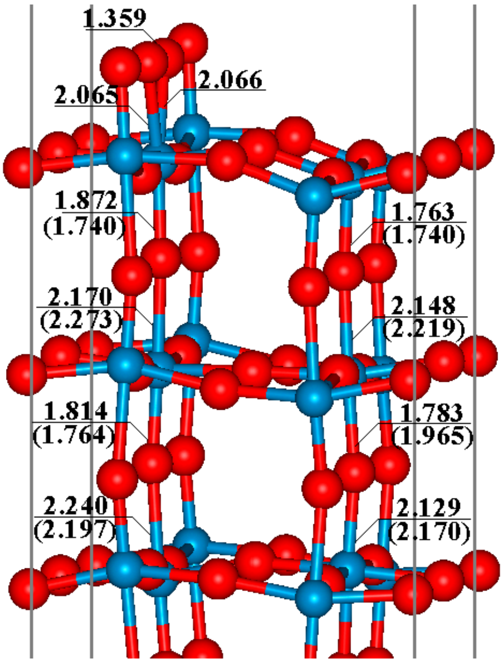

-0.628eV

(e)

Figure S1. Possible adsorption configurations for O_2 adsorption on the defective $WO_3(001)$ surface. The bond lengths (Å) close to the adsorption site are given, and the data in the parenthesis are in relative to the defective $WO_3(001)$ surface before O_2 adsorption. In addition, the corresponding adsorption energies have also been provided.
